# Direct Oral Anticoagulants Versus Aspirin for Stroke Prevention After Embolic Stroke of Undetermined Source: An Updated Meta-Analysis of Randomized Controlled Trials

**DOI:** 10.3390/jcm13226730

**Published:** 2024-11-08

**Authors:** Edoardo Pirera, Lucio D’Anna, Domenico Di Raimondo, Antonino Tuttolomondo

**Affiliations:** 1 Internal Medicine and Stroke Care Ward, Department of Promoting Health, Maternal-Infant, Excellence and Internal and Specialized Medicine (Promise) G. D’Alessandro, University of Palermo, 90133 Palermo, Italy; edoardo.pirera@unipa.it (E.P.); domenico.diraimondo@unipa.it (D.D.R.); bruno.tuttolomondo@unipa.it (A.T.); 2 Department of Stroke and Neuroscience, Charing Cross Hospital, Imperial College London NHS Healthcare Trust, London W2 1NY, UK; 3 Department of Brain Sciences, Imperial College London, London SW7 2AZ, UK

**Keywords:** embolic stroke of undetermined source, direct oral anticoagulation, ESUS, DOAC

## Abstract

**Background**: Four randomized controlled trials (RCTs) did not show a benefit of direct oral anticoagulant (DOAC) treatment compared with antiplatelet therapy for the prevention of recurrent strokes in patients with embolic stroke of undetermined source (ESUS). However, the balance between efficacy and safety in subgroups needs to be better defined. We aimed to assess the relative benefits of DOACs in key subgroups of adult patients with ESUS. **Methods**: We searched major databases (PubMed, Embase, CENTRAL, and Web of Science) for RCTs published from inception to 16 June 2024. The primary outcome was recurrent stroke, and the main safety outcomes were major bleeding and clinically relevant non-major bleeding (CRNB). We assessed the risk of bias using the Cochrane Risk of Bias tool 2. **Results**: We identified four RCTs, involving a total of 13,970 patients with ESUS. Compared to antiplatelet therapy, treatment with DOAC did not reduce the risk of recurrent stroke (RR 0.95, 95% CI 0.83–1.08, *p* = 0.45) or ischemic stroke (RR 0.92, 95% CI 0.80–1.05, *p* = 0.22) or increase major bleeding (RR 1.57, 95% CI 0.87–2.83; *p* = 0.14). DOAC treatment was associated with a significantly higher risk of CRNMB compared to aspirin (RR 1.52, 95% CI: 1.22–1.90; *p* = 0.0002). The subgroup analysis demonstrated that use of DOACs was associated with a significant protective effect in patients aged 75 or older (RR 0.76, 95% CI 0.60–0.97, *p* = 0.03) and when the time from index stroke to randomization was ≥8 days (RR 0.80, 95% CI 0.66–0.97, *p* = 0.02) in preventing recurrency of any type of stroke. **Conclusions**: Our meta-analysis showed lack of overall benefit of anticoagulation with DOAC compared to antiplatelet therapy for recurrent stroke prevention in adult patients with ESUS. However, the subgroup analyses suggest the possibility of interactions between age and timing of randomization since stroke and treatment with an DOAC in terms of recurrent stroke prevention. Further research toward tailoring the antithrombotic strategy according to patient characteristics is needed.

## 1. Introduction

In 2014, the term embolic stroke of undetermined source (ESUS) was introduced to describe patients with a non-lacunar ischemic stroke and no convincing etiology [1]. ESUS and cryptogenic stroke are not synonyms, as the former represents an etiologically heterogenous group and may be caused by various potential sources of thromboembolism while the latter includes patients with multiple stroke etiologies or incomplete diagnostic work-up [2]. ESUS represents a large patient group as it involves approximately 17% of all ischemic stroke patients, who are typically younger patients with mild strokes [3]. Covert atrial fibrillation (AF) was conceived as the most important mechanism in ESUS patients as randomized controlled trials (RCTs) of prolonged cardiac monitoring, meta-analyses, and observational studies showed that AF may be detected in up to 30% of ESUS patients during long-term follow-up [4,5,6,7,8]. However, a pooled analysis of registry data showed that ESUS patients have a considerable rate of stroke recurrence of 4% to 5% after a year even though these patients were largely treated with antiplatelets [3]. Therefore, it was evident that new antithrombotic strategies were needed to reduce the risk of recurrent strokes in ESUS patients.

Four large RCTs, NAVIGATE ESUS (Rivaroxaban Versus Aspirin in Secondary Prevention of Stroke and Prevention of Systemic Embolism in Patients With Recent Embolic Stroke of Undetermined Source) [9], RE-SPECT ESUS (Dabigatran Etexilate for Secondary Stroke Prevention in Patients With Embolic Stroke of Undetermined Source) [10], ATTICUS (Apixaban for the Treatment of Embolic Stroke of Undetermined Source) [11], and ARCADIA (Apixaban to Prevent Recurrence After Cryptogenic Stroke in Patients with Atrial Cardiopathy) [12], concluded that direct oral anticoagulants (DOACs) were not associated with lower rates of stroke recurrence compared with aspirin in ESUS patients. A previous meta-analysis of only two RCTs, NAVIGATE and RE-SPECT ESUS [9,10], has shown that DOACs are not more effective than aspirin in preventing stroke recurrence in patients with ESUS and they increase the risk of bleeding [13]. More recently, Marinheiro et al. conducted a meta-analysis including all four RCTs [14]. The authors concluded that in patients with ESUS, DOACs did not reduce the risk of recurrent stroke, ischemic stroke, or systemic embolism, or all-cause mortality. Furthermore, their age and sex subgroup analysis showed no significant differences between groups. However, the authors seem to have misreported the number of events in the age group ≥75 years old due to a misinterpretation of the NAVIGATE ESUS trial’s age subgroup categories. This led to the exclusion of 11 events, which has potentially impacted on the results of this analysis.

We, therefore, conducted a new systematic review and updated meta-analysis, including all four available RCTs, to thoroughly investigate the efficacy and safety of oral anticoagulation compared with antiplatelet therapy for secondary stroke prevention in adult patients with ESUS. In addition, to address the limitations of Marinheiro et al.’s previous meta-analysis [14], our study also places particular emphasis on a more comprehensive key patient subgroup analysis. Through more detailed subgroup analyses, we aim to offer a deeper and more precise understanding of the outcomes within distinct patient populations.

## 2. Materials and Methods

### 2.1. Search Strategy, Selection Criteria, and Data Extraction

We systematically searched for peer-reviewed reports without language restrictions in PubMed, Embase, WebOfScience, Cochrane Central Register of Controlled Trials (CENTRAL), and Cochrane Reviews from inception to 16 June 2024. The search term strategy included a combination of synonyms for “ESUS” and “DOAC” alongside “apixaban/dabigatran/edoxaban/rivaroxaban”. The complete search strategy is available in Supplementary S1 in the Supplementary Materials. This systematic review and meta-analysis was registered on the International Prospective Register of Systematic Reviews (PROSPERO, CRD42024568058) and reported in accordance with the Preferred Reporting Items for Systematic Review and Meta-Analysis (PRISMA) statement guidelines. The PRISMA-S checklist is available in Supplementary S2 in the Supplementary Materials. For the purpose of this meta-analysis, we included studies that met all the following eligibility criteria: (1) RCTs; (2) comparison between DOAC and aspirin; (3) carried out in patients who had received a diagnosis of ESUS; and (4) reporting at least one of the clinical outcomes of interest. We excluded studies with (1) overlapping patient populations and (2) without a placebo control group. Editorials, letters to editors, conference abstracts, or any studies that have not been peer-reviewed were excluded. Two authors (EP and LDA) independently screened the titles and abstracts of potential records identified in the literature search. Subsequently, they independently reviewed the full text of any records deemed potentially eligible based on the pre-defined eligibility criteria. Disagreements were resolved by reaching a consensus with a third reviewer (DDR).

### 2.2. Data Extraction, Endpoint Definition and Subgroup Analysis

Data extraction was independently performed by two authors (EP and DDR) and reported on an electronic spreadsheet. Disagreements during the data extraction were resolved through discussion between the two authors. In cases of persistent disagreement, a third reviewer provided a final opinion to reach consensus (AT). The primary efficacy outcome of interest was recurrent stroke, including ischemic, hemorrhagic, or undefined type. Secondary efficacy outcomes were the following: (1) recurrent ischemic stroke; (2) recurrent hemorrhagic stroke, (3) systemic embolism; (4) Major Adverse Cardiovascular Events (MACE) defined as a composite of non-fatal stroke, non-fatal myocardial infarction or death from cardiovascular event; (5) death from “all-cause”; (6) death from cardiovascular causes. The safety outcomes were (1) major bleeding (MB) defined according to the “International Society on Thrombosis and Hemostasis” (ISTH) criteria [15]; (2) clinically relevant non-major bleeding (CRNMB) events defined as hemorrhagic events that do no meet the criteria for MB and require hospital admission or medical/surgical treatment; (3) a composite of MB or CRNMB; (4) intracranial hemorrhage; or (5) life-threatening or fatal bleeding defined as a subgroup of major bleeds with reduction in hemoglobin of at least 5 g/dL, symptomatic intracranial bleeding, the transfusion of at least four units of packed red blood cells, associated with hemodynamic instability requiring the use of intravenous inotropic agents, or requiring surgical intervention. Prespecified subgroup analysis for the primary outcomes included age, sex, time from index stroke to randomization, CHA2DS2-VASc score [16], history of transient ischemic attack (TIA) or stroke, history of arterial hypertension, history of diabetes mellitus, and patent foramen ovale (PFO).

### 2.3. Risk of Bias Assessment

A quality assessment of individual studies was conducted by EP using the “Risk of Bias 2” (RoB 2) tool wherein studies are scored as low, some concern, or a high risk of bias in 5 domains: randomisation process, deviation from intended intervention, missing data, measurement of the outcome and selection of the reported result [17].

### 2.4. Statistical Analysis

Treatment effects for dichotomous outcomes were compared using pooled risk ratios (RRs) with 95% confidence intervals. Heterogeneity was evaluated with the Cochrane Q test and I^2^ statistics with *p* values inferior to 0.10 and I^2^ > 40% were considered significant for heterogeneity, respectively. Furthermore, according to the Cochrane collaboration, we considered different degree of heterogeneity as follows: (1) an I^2^ statistic with a value of 0–40% indicates a low heterogeneity; (2) 30–60% represents moderate heterogeneity; (3) 50–90% indicates substantial heterogeneity; and (4) 75–100% indicates considerable heterogeneity [18]. We used a fixed-effect model for endpoints with I^2^ ≤ 40% (low heterogeneity); otherwise, the DerSimonian and Laird random-effects model was used. To evaluate the robustness and consistency of the results, a sensitivity analysis was conducted using a Jackknife (leave-one-out) method for meta-analysis with at least three studies [19]. This method systematically excluded individual studies and recalculated pooled estimates to ascertain the influence of any single study on the overall results. RevMan Web (Nordic Cochrane centre, The Cochrane collaboration, Copenhagen, Denmark) was used for statistical analysis. Sensitivity analysis was performed using R 4.4.1 (Foundation for Statistical Computing, Vienna, Austria).

## 3. Results

**Figure 1:**
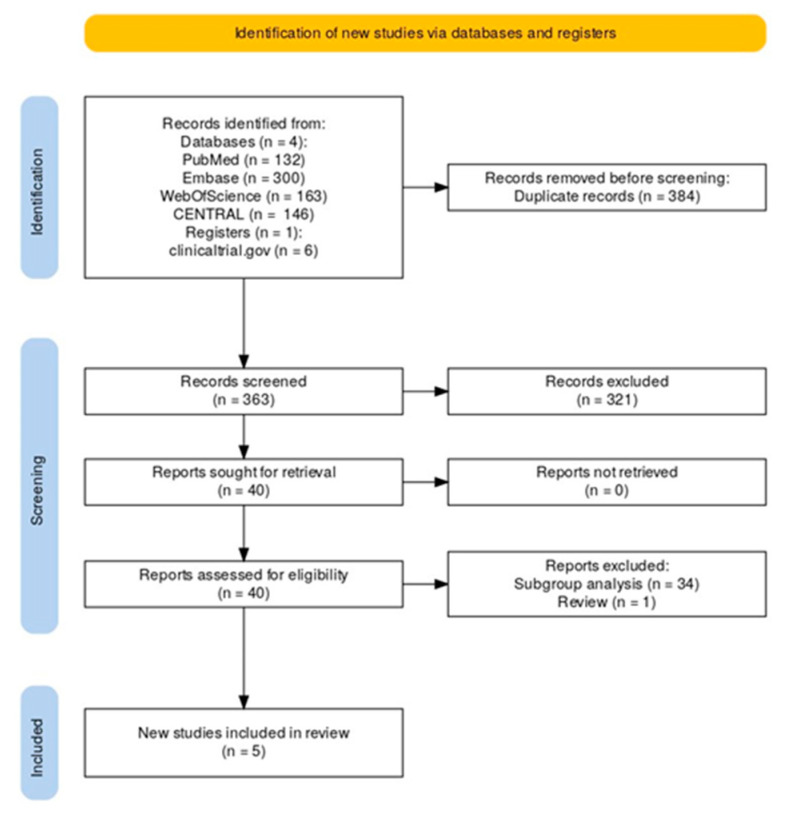
PRISMA flow diagram of study screening and selection.

The initial literature search yielded 747 results. Following the removal of 384 duplicates, 323 records were excluded based on title and abstract inspection. A full-text review was conducted for forty articles, ultimately resulting in the inclusion of five studies in the quantitative analysis. Figure 1 shows the PRISMA flow diagram of the systematic literature search, study selection, and reasons for exclusion. Four studies were RCTs [9,10,11,12] and one study was a previous systematic review and meta-analysis of two included RCTs [13]. The four RCTs included in the meta-analysis included a total of 13,970 patients with a pooled mean age of 65.96 years and comprised mostly men (60.73%). Regarding comorbidities, 76.2% of participants had hypertension and 17.8% had a previous history of stroke/TIA. Additionally, 20.4% reported a previous history of or active smoking and 24.5% had diabetes. Table 1 presents the main characteristics of the included studies. For RE-SPECT ESUS, ATTICUS, and ARCADIA, ESUS was defined according to the “Cryptogenic Stroke/ESUS Internation Working Group” criteria [3]: (1) stroke detected by computed tomography or magnetic resonance imaging that is not lacunar; (2) absence of extracranial or intracranial atherosclerosis causing ≥ 50% luminal stenosis according to North American Symptomatic Carotid Endarterectomy Trial (NASCET) [20,21] in arteries supplying the ischemic area; (3) no major-risk cardioembolic source of embolism; (4) no other specific cause of stroke identified. The definition of ESUS in the NAVIGATE trial contrasted with the “Cryptogenic Stroke/ESUS Internation Working Group” criteria in three main ways: (1) intracranial arterial imaging is not required; (2) intracranial arterial occlusion does not exclude inclusion in the trial if diagnosed as embolic; and (3) exclusion based on echocardiography was limited to intracardiac thrombus [22]. Additionally, in the ATTICUS trial [11], participants had to have at least one clinical, electrocardiographic, or echocardiographic “enriched factor predictive for AF”: (1) CHA_2_DS_2_-VASc score greater than or equal to four; (2) atrial high-rate episodes; (3) left atrial size greater than 45 mm, spontaneous echo contrast, or flow velocity less than or equal to 0.2 m/s in the left atrial appendage; (4) PFO [23]. Notably, ATTICUS [11] and ARCADIA [12] were prematurely stopped for futility without any safety concerns after the planned interim analysis. According to the RoB 2 tool from Cochrane [17], all RCTs included in the meta-analysis were deemed to have a low risk of bias, indicating the high-quality evidence in the studies (Supplementary S3).

### 3.1. Results of Meta-Analysis for the Primary Efficacy Outcome

**Figure 2:**
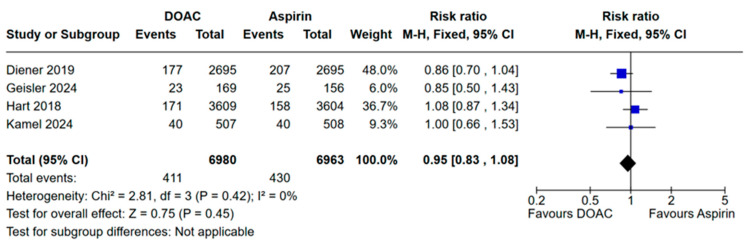
Forest plot showing the pooled estimates of risk ratio and the 95% CI for the primary efficacy outcome “recurrent stroke” [9,10,11,12].

Recurrent stroke occurred in 411 patients in the DOAC group (5.88%) and in 430 patients in the aspirin group (6.17%). A meta-analysis of the pooled events showed no statistically significant difference between DOACs and aspirin in preventing recurrent stroke: pooled RR: 0.95, 95% CI 0.83–1.08, I^2^: 0%; test for overall effect: *p* = 0.45; Cochran’s Q test: *p* = 0.42 (Figure 2). When individually removing each study, the results remained consistent with the main analysis (Supplementary S4).

### 3.2. Results of Meta-Analysis for the Secondary Efficacy Outcomes

Treatment with DOACs showed no statistically significant differences compared to antiplatelet therapy in several of the key outcomes examined. The risk of ischemic stroke was not significantly reduced (RR 0.92, 95% CI 0.80–1.05, I^2^: 0%; test for overall effect: *p* = 0.22; Cochran’s Q test: *p* = 0.70; Supplementary S5A). Similarly, no significant differences were observed for hemorrhagic stroke (RR 2.21, 95% CI 0.29–16.69, I^2^: 79%; test for overall effect: *p* = 0.44; Cochran’s Q test: *p* = 0.03; Supplementary S5B), systemic embolism (RR 0.52, 95% CI 0.21–1.24, I^2^: 0%; test for overall effect: *p* = 0.14; Cochran’s Q test: *p* = 0.95; Supplementary S5C), myocardial infarction (RR 0.93, 95% CI 0.61–1.42, I^2^: 16%; test for overall effect: *p* = 0.74; Cochran’s Q test: *p* = 0.30; Supplementary S5D), all-cause mortality (RR 1.11, 95% CI 0.87–1.42, I^2^: 0%; test for overall effect: *p* = 0.38; Cochran’s Q test: *p* = 0.68; Supplementary S5E), or death from cardiovascular events (RR 1.12, 95% CI 0.76–1.66, I^2^: 15%; test for overall effect: *p* = 0.56; Cochran’s Q test: *p* = 0.31; Supplementary S5F). Furthermore, the composite endpoint of non-fatal stroke, non-fatal myocardial infarction, and cardiovascular death did not show a significant reduction (RR 0.96, 95% CI 0.85–1.10, I^2^: 0%; test for overall effect: *p* = 0.58; Cochran’s Q test: *p* = 0.41; Supplementary S5G). The leave-one-out analysis showed that the results from the meta-analyses for the secondary outcomes were generally robust and remained consistent with the pooled analysis (Supplementary S6).

### 3.3. Results of Meta-Analysis for the Primary Safety Outcomes

**Figure 3:**
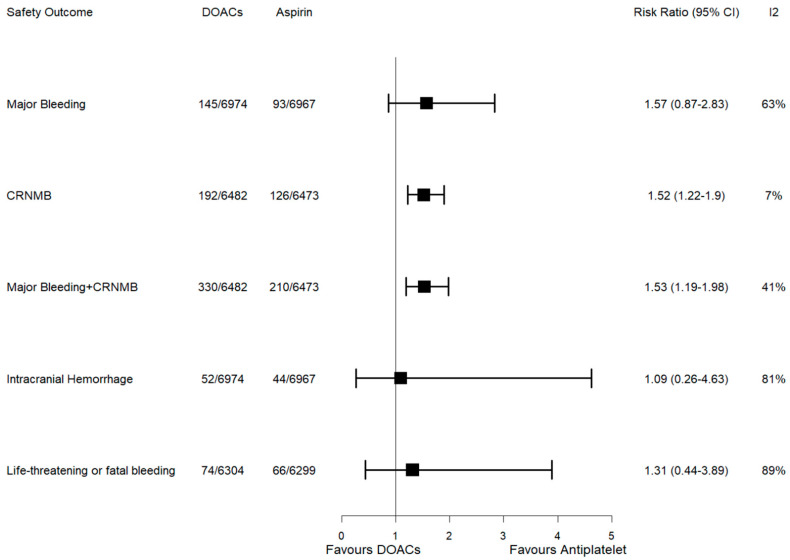
Forest plot for safety outcomes. CRNMB: clinically relevant non-major bleeding; DOACs: direct oral anticoagulants; 95% CI: 95% confidence interval.

Data from the four RCTs were used for pooling MB events. MB occurred in 145 patients in the DOAC group (2.07%) and in 93 patients in the aspirin group (1.33%). Compared to aspirin, DOACs showed no increase in the risk of MB events (pooled RR 1.57, 95% CI 0.87–2.83; test for overall effect: *p* = 0.14; Cochran’s Q test: *p* = 0.04) with substantial heterogeneity among studies (I^2^: 63%). Three studies [9,10,11] (12,955 participants) reported data for CRNMB events. CRNMB occurred in 192 patients in the DOAC group (2.96%) and in 126 patients in the aspirin group (1.94%). DOACs were associated with a significantly higher risk of CRNMB compared to aspirin (RR 1.52, 95% CI: 1.22–1.90; test for overall effect: *p* = 0.0002; Cochran’s Q test: *p* = 0.34) with a low heterogeneity among the studies (I^2^: 7%). A meta-analysis of the data from RE-SPECT ESUS, NAVIGATE, and ATTICUS [9,10,11] for the composite of MB and CRNMB showed a significantly increased risk with DOACs compared to aspirin (RR 1.53, 95% CI: 1.19–1.98; Cochran’s Q test: *p* = 0.18). MB and CRNMB occurred in 330 patients in the DOAC group (5.09%) and in 210 patients in the aspirin group (3.24%). The overall effect was statistically significant (*p* = 0.001), although with moderate heterogeneity (I²: 41%). Intracranial hemorrhage occurred in 52 patients in the DOAC group (0.74%) and in 44 patients in the aspirin group (0.63%) with a pooled RR 1.09 (95% CI 0.26–4.63; test for overall effect: *p* = 0.9; Cochran’s Q test: *p* = 0.006; I^2^: 81%). Finally, life-threatening or fatal bleeding occurred in 74 patients in the DOAC group (1.17%) and in 66 patients in the aspirin group (1.04%) with a pooled RR 1.31 (95% CI 0.44–3.89, test for overall effect: *p* = 0.63; Cochran’s Q test: *p* = 0.003; I^2^: 89%). In the sensitivity analysis for MB, statistical significance was achieved upon removal of RE-SPECT ESUS [10]. Conversely, for the composite outcome of MB and CRNMB, the statistical significance was lost when NAVIGATE [9] and RE-SPECT ESUS [10] were individually removed from the analysis. In contrast, for CRNMB and intracranial hemorrhage, the sensitivity analysis confirmed the robustness of our findings (Supplementary S7). The results of meta-analyses for primary safety outcomes are shown in Figure 3.

### 3.4. Results of Subgroup Analysis for Recurrent Stroke

**Figure 4:**
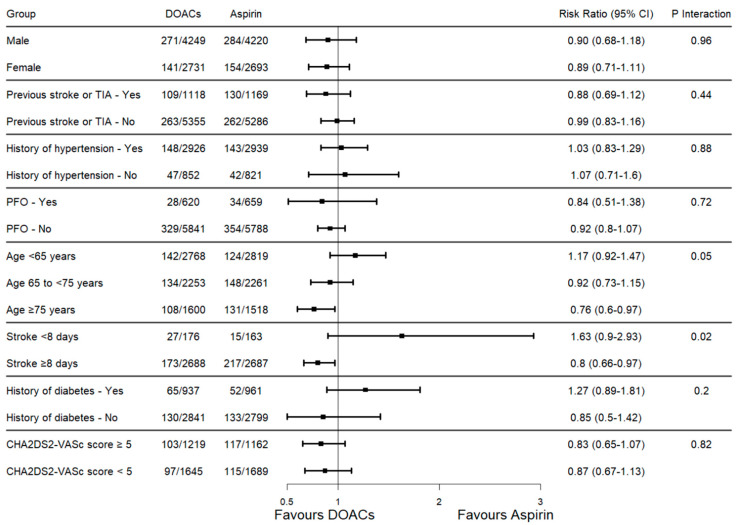
Forest plot of subgroup analysis for the primary efficacy outcome “recurrent stroke”. TIA: transient ischemic stroke; PFO: patent foramen ovale; DOACs: DOACs: direct oral anticoagulants; 95% CI: 95% confidence interval.

The results of a subgroup analysis are shown in Figure 4. Subgroup analyses according to sex, history of TIA or stroke, hypertension, diabetes, and presence of PFO showed no difference between DOACs and aspirin in preventing the recurrence of any type of stroke. The same results were observed for a subset of patients with a CHA_2_DS_2_-VASc score ≥ 5 compared to those with a score lower than 5. Subgroup analyses according to different age groups and the time from index stroke to randomization to treatment with DOACs or aspirin suggested a potential benefit of the anticoagulants compared to aspirin. Specifically, three age subgroups were analyzed: (1) <65 years with no significant difference between DOACs and aspirin (pooled RR 1.17, 95% CI 0.92–1.47, test for overall effect: *p* = 0.2; Cochran’s Q test: *p* = 0.36; I^2^ = 1%); (2) 65 to <75 years with no significant difference (pooled RR 0.92, 95% CI 0.73–1.15, test for overall effect: *p* = 0.46; Cochran’s Q test: *p* = 0.39; I^2^ = 0%), and (3) ≥75 years where DOACs showed a significant protective effect (pooled RR 0.76, 95% CI 0.60–0.97, test for overall effect: *p* = 0.03; Cochran’s Q test: *p* = 0.24; I^2^ = 29%). The test for subgroup differences approached significance (*p* interaction = 0.05, Cochran’s Q test: *p* = 0.05; I^2^ = 67.7%), suggesting a potential age-dependent efficacy of DOACs compared to aspirin in preventing any recurrent stroke after an ESUS. Regarding the analysis according to the time from index stroke to randomization, for the early period (<8 days), DOACs showed no significant difference compared to aspirin (pooled RR 1.63, 95% CI 0.90–2.93, test for overall effect: *p* = 0.1; Cochran’s Q test: *p* = 0.25; I^2^ = 24%). In contrast, for the later period (≥8 days), DOACs demonstrated a significant protective effect (pooled RR 0.80, 95% CI 0.66–0.97, test for overall effect: *p* = 0.02; Cochran’s Q test: *p* = 0.54; I^2^ = 0%). The test for subgroup differences was significant (*p* interaction = 0.02, Cochran’s Q test: *p* = 0.02; I^2^ = 80.5%), suggesting a time-dependent effect of DOACs versus aspirin. These findings indicate that the efficacy of DOACs versus aspirin may vary based on time from initiation after the index ESUS and patient age, with potentially greater benefits in later treatment phases and older patients. No data were available for bleeding events in these subgroups analyzed. The sensitivity analysis showed the robustness of our findings except for the meta-analysis of subjects ≥75 years that lost statistical significance when individually removing the results of RE-SPECT ESUS [10] and ATTICUS [11] from the main analysis (Supplementary S8).

## 4. Discussion

Our meta-analysis of the four RCTs available showed that anticoagulation with DOACs is not superior compared to antiplatelet therapy for recurrent stroke prevention in adult patients with ESUS. We provided evidence that there is no benefit of anticoagulation in any of the secondary efficacy outcomes investigated including risk of any ischemic stroke, systemic embolism, myocardial infarction, and all cause of mortality. In terms of safety outcomes, our analysis indicated that anticoagulation with DOAC significantly increased the risk of CRNMB, composite-outcome MB, or CRNMB, while, compared to aspirin, the anticoagulants did not significantly increase the risk of intracranial hemorrhage and life-threatening or fatal bleeding in patients with ESUS. These results are largely in line with the results of the previous meta-analysis of the first two RCTs, NAVIGATE ESUS and RE-SPECT ESUS, by Hariharan et al. [13] that showed a lack of overall benefit of anticoagulation compared with antiplatelet therapy in patients with ESUS. A recent meta-analysis on randomized controlled trials of patients with ESUS by Ghannam et al. [24] has also documented that the empiric anticoagulation approach is not beneficial for patients with ESUS. Our results have largely confirmed the above findings.

However, one of the main findings of our subgroup analysis is that we showed a benefit of anticoagulation in ESUS patients in terms of recurrent stroke prevention when the time delay from the index event to the randomization was 8 days or over regardless of the severity or volume of the acute ischemic stroke. Conversely, our subgroup analyses also suggest that aspirin might have a benefit during the first eight days after stroke although this finding is not statistically significant. This result was based on the data from the ATTICUS [11] and RE-SPECT ESUS [10] trials where subgroup analyses suggested the possibility of an interaction between treatment and the timing of randomization since stroke, although this did not reach the level of significance. Of note, we did not include data from the NAVIGATE ESUS [9] trial in this sub-analysis as the time from the index event to stroke randomization was set at different and not comparable time points. The risk of both recurrent ischemic stroke and intracranial hemorrhage is highest in the first few days after the acute ischemic stroke, and the evidence showed larger benefit of using aspirin in the immediate period after stroke [25]. Further studies are warranted to confirm our findings.

Furthermore, our analysis suggests a potential benefit of using direct oral anticoagulants (DOACs) over aspirin in the prevention of recurrent stroke among patients with embolic stroke of undetermined source (ESUS) aged 75 years or older. This finding aligns with subgroup analyses from the RE-SPECT ESUS trial, which demonstrated a significant benefit of lower-dose dabigatran compared to aspirin in patients aged over 75. However, this observation contrasts with the findings of Ghannam et al. [24], who reported no significant difference between the two treatment modalities in age-stratified subgroups. It is important to note that Ghannam et al. employed random-effects models, whereas our meta-analysis utilized a fixed-effect model. While random-effects models are frequently preferred in meta-analyses due to their ability to account for between-study heterogeneity and are often considered more conservative, several studies have indicated that this is not universally true [26]. Therefore, further research is needed to clarify the role of anticoagulation therapy in age-defined subgroups of ESUS patients.

Besides covert AF, the causes of ESUS are heterogenous and they encompass proximal embolic sources (e.g., atrial myopathy, PFO, left ventricular disease) and also supracardiac atherosclerotic plaques and non-stenotic extracranial carotid plaques that overall might respond better to antiplatelet treatment rather than to DOACs [3,27]. Indeed, Ntaios et al. performed an exploratory sub-analysis that included 1382 patients from the NAVIGATE ESUS RCT to evaluate the rate of aortic arch atherosclerosis, their characteristics and response to treatment [28]. In this exploratory analysis the authors found that, among the entire cohort of ESUS patients who were assessed with a transesophageal echocardiography, 29% had aortic arch atherosclerosis and 8% had complex aortic arch atherosclerosis defined as being ulcerated or more than 4 mm in thickness or had a mobile thrombus present. Age, diabetes mellitus, coronary artery disease, aortic valvulopathy, and statin use before randomization were all associated with complex aortic arch atherosclerosis, all of which indicate a high atherosclerotic burden. The high prevalence of aortic arch atherosclerosis in ESUS patients might suggest that ESUS patients with high-risk aortic arch atherosclerosis may represent a unique atherosclerotic subgroup that could benefit from tailored medical therapy to target arterial atherosclerosis. Furthermore, the etiologic role of carotid atherosclerotic plaques in patients with ESUS was assessed in several recent studies [29,30,31,32]. In the NAVIGATE ESUS trial and in other studies, carotid plaque was more often present ipsilateral to the qualifying ischemic stroke than contralateral. In addition, in the AF-ESUS study, new incident AF was less frequently detected in ESUS patients with ipsilateral carotid plaques compared with those without [33]. Similarly, a strong negative association was reported between carotid plaques and PFO in young adults with cryptogenic stroke [34]. These data are important in selecting appropriate therapy options in ESUS patients and further studies investigating the role of carotid endarterectomy versus intensive medical therapy alone might be warranted to determine the optimal secondary stroke prevention strategy in this subgroup of ESUS patients.

### Limitations

This meta-analysis has several limitations that warrant consideration. First, although the four trials included in this analysis demonstrated a low risk of bias, this limited number could impact the generalizability of our results. Second, the ATTICUS trial focused on a specific ESUS subpopulation with an “enriched factor predictive for AF”. However, this subgroup represented a small proportion of the overall meta-analysis population. Third, sensitivity analyses showed that certain meta-analysis results lacked robustness, requiring cautious interpretation. Furthermore, it is important to acknowledge that several subgroups were identified post hoc, based on the availability of published data. Consequently, the subgroup analyses should be regarded as exploratory and hypothesis-generating rather than confirmatory. Because we did not have individual participant data for all the trials, our statistical approach was carried out at the study level. Fourth, the absence of comprehensive bleeding data across all subgroups and the lack of access to individual patient data posed a limitation. A more detailed analysis using individual patient data could have provided greater statistical power for examining subgroups and allowed for a more informed assessment of the risk–benefit profile of DOACs compared to aspirin in the ESUS population. Finally, we acknowledge that the use of a fixed-effect model may have resulted in an underestimation of the small number of studies included in the analysis. However, it should be noted that we used such model only for meta-analyses with non-significant heterogeneity (Cochran’s Q test < 0.10 or I^2^ > 40%).

## 5. Conclusions

Our meta-analysis showed a lack of an overall benefit of using anticoagulation with DOACs compared to antiplatelet therapy for recurrent stroke prevention in adult patients with ESUS. Our analysis also indicated that anticoagulation with DOACs significantly increased the risk of CRNMB, composite-outcome MB, or CRNMB in patients with ESUS. However, the subgroup analyses suggest the possibility of interactions between age and timing of randomization since stroke with treatment with DOAC in terms of recurrent stroke prevention. Further research toward tailoring the antithrombotic strategy according to patient characteristics is needed.

## Figures and Tables

**Table 1:** Characteristics of studies included in the meta-analysis.

Study, Year and Reference	Identifier	Design	Total Population (*n*)	Intervention	Control Group	Age (Year ± SD)	Median Follow-Up
Hart, 2018 [9]	NAVIGATE, NCT02313909	RCT	7213	Rivaroxaban 15 mg OD	Aspirin 100 mg OD	66.9 ± 9.8	11 months
Diener, 2019 [10]	RE-SPECT ESUS, NCT02239120	RCT	5390	Dabigatran 150 mg BID Or Dabigatran 110 mg BID	Aspirin 100 mg OD	64.2 ± 11.4	19 months
Geisler, 2024 [11]	ATTICUS, NCT02427126	RCT	352	Apixaban 5 mg BID Or Apixaban 2.5 mg BID	Aspirin 100 mg OD	68.4 ± 10.4	N/A
Kamel, 2024 [12]	ARCADIA, NCT03192215	RCT	1015	Apixaban 5 mg BID Or Apixaban 2.5 mg BID	Aspirin 81 mg OD	68.0 ± 10.9	1.8 years

RCT: randomized controlled trial; OD: once die; mg: milligrams; BID: bis in die; SD: standard deviation; N/A: not available.

## Supplementary Materials

The following supporting information can be downloaded at: https://www.mdpi.com/article/10.3390/jcm13226730/s1. Reference [35] is cited in the supplementary materials.
