# Supplementary material for: Direct Oral Anticoagulants Versus Aspirin for Stroke Prevention After Embolic Stroke of Undetermined Source: An Updated Meta-Analysis of Randomized Controlled Trials
Source: J Clin Med. 2024 Nov 8;13(22):6730. doi: 10.3390/jcm13226730 (PMC11595132; doi:10.3390/jcm13226730)
Supplement: Supplementary file 1 [file jcm-13-06730-s001.zip › jcm-3243076-supplementary.pdf]

Supplementary S1. Full electronic search for PubMed/MEDLINE

((“Embolic Stroke of Undetermined Source”[tiab]) OR (“cryptogenic stroke”[tiab]) OR (“ESUS”[tiab])) AND ((“DOAC”[tiab]) OR (“Direct Factor Xa Inhibitor*”[tiab]) OR (“oral anticoagulant*”[tiab]) OR (“Direct Acting Oral Anticoagulant*”[tiab]) OR (“Direct-Acting Oral Anticoagulant*”[tiab]) OR (“oral anticoagulat*”[tiab]) OR (“direct oral anticoagulant*”[tiab]) OR (“new oral anticoagulant*”[tiab]) OR (“dabigatran”[tiab]) OR (“apixaban”[tiab]) OR (“edoxaban”[tiab]) OR (“rivaroxaban”[tiab])) AND (“randomized controlled trial”[pt] OR “controlled clinical trial”[pt] OR “randomized”[tiab] OR “placebo”[tiab] OR “drug therapy”[sh] OR “randomly”[tiab] OR “trial”[tiab] OR “groups”[tiab])

Supplementary S2: PRISMA-S checklist [1]

Section and Topic	Item #	Checklist item	Location where item is reported
**TITLE**			
Title	1	Identify the report as a systematic review.	Title page
**ABSTRACT**			
Abstract	2	See the PRISMA 2020 for Abstracts checklist.	2-3
**INTRODUCTION**			
Rationale	3	Describe the rationale for the review in the context of existing knowledge.	4-5
Objectives	4	Provide an explicit statement of the objective(s) or question(s) the review addresses.	5
**METHODS**			
Eligibility criteria	5	Specify the inclusion and exclusion criteria for the review and how studies were grouped for the syntheses.	5
Information sources	6	Specify all databases, registers, websites, organisations, reference lists and other sources searched or consulted to identify studies. Specify the date when each source was last searched or consulted.	5
Search strategy	7	Present the full search strategies for all databases, registers and websites, including any filters and limits used.	Supplementary Appendix, page 1
Selection process	8	Specify the methods used to decide whether a study met the inclusion criteria of the review, including how many reviewers screened each record and each report retrieved, whether they worked independently, and if applicable, details of automation tools used in the process.	5-6
Data collection process	9	Specify the methods used to collect data from reports, including how many reviewers collected data from each report, whether they worked independently, any processes for obtaining or confirming data from study investigators, and if applicable, details of automation tools used in the process.	6
Data items	10a	List and define all outcomes for which data were sought. Specify whether all results that were compatible with each outcome domain in each study were sought (e.g. for all measures, time points, analyses), and if not, the methods used to decide which results to collect.	6
	10b	List and define all other variables for which data were sought (e.g. participant and intervention characteristics, funding sources). Describe any assumptions made about any missing or unclear information.	6
Study risk of bias assessment	11	Specify the methods used to assess risk of bias in the included studies, including details of the tool(s) used, how many reviewers assessed each study and whether they worked independently, and if applicable, details of automation tools used in the process.	6-7
Effect measures	12	Specify for each outcome the effect measure(s) (e.g. risk ratio, mean difference) used in the synthesis or presentation of results.	7
Synthesis methods	13a	Describe the processes used to decide which studies were eligible for each synthesis (e.g. tabulating the study intervention characteristics and comparing against the planned groups for each synthesis (item #5)).	7
	13b	Describe any methods required to prepare the data for presentation or	7

Section and Topic	Item #	Checklist item	Location where item is reported
		synthesis, such as handling of missing summary statistics, or data conversions.	
	13c	Describe any methods used to tabulate or visually display results of individual studies and syntheses.	7
	13d	Describe any methods used to synthesize results and provide a rationale for the choice(s). If meta-analysis was performed, describe the model(s), method(s) to identify the presence and extent of statistical heterogeneity, and software package(s) used.	7
	13e	Describe any methods used to explore possible causes of heterogeneity among study results (e.g. subgroup analysis, meta-regression).	7
	13f	Describe any sensitivity analyses conducted to assess robustness of the synthesized results.	7
Reporting bias assessment	14	Describe any methods used to assess risk of bias due to missing results in a synthesis (arising from reporting biases).	N/A
Certainty assessment	15	Describe any methods used to assess certainty (or confidence) in the body of evidence for an outcome.	7
**RESULTS**			
Study selection	16a	Describe the results of the search and selection process, from the number of records identified in the search to the number of studies included in the review, ideally using a flow diagram.	7
	16b	Cite studies that might appear to meet the inclusion criteria, but which were excluded, and explain why they were excluded.	N/A
Study characteristics	17	Cite each included study and present its characteristics.	7-8
Risk of bias in studies	18	Present assessments of risk of bias for each included study.	8
Results of individual studies	19	For all outcomes, present, for each study: (a) summary statistics for each group (where appropriate) and (b) an effect estimate and its precision (e.g. confidence/credible interval), ideally using structured tables or plots.	8-11
Results of syntheses	20a	For each synthesis, briefly summarise the characteristics and risk of bias among contributing studies.	8
	20b	Present results of all statistical syntheses conducted. If meta-analysis was done, present for each the summary estimate and its precision (e.g. confidence/credible interval) and measures of statistical heterogeneity. If comparing groups, describe the direction of the effect.	8-11
	20c	Present results of all investigations of possible causes of heterogeneity among study results.	8-11
	20d	Present results of all sensitivity analyses conducted to assess the robustness of the synthesized results.	8-11
Reporting biases	21	Present assessments of risk of bias due to missing results (arising from reporting biases) for each synthesis assessed.	N/A
Certainty of evidence	22	Present assessments of certainty (or confidence) in the body of evidence for each outcome assessed.	8-11
**DISCUSSION**			
Discussion	23a	Provide a general interpretation of the results in the context of other evidence.	11-15
	23b	Discuss any limitations of the evidence included in the review.	14-15
	23c	Discuss any limitations of the review processes used.	
	23d	Discuss implications of the results for practice, policy, and future research.	11-15
**OTHER INFORMATION**			
Registration and protocol	24a	Provide registration information for the review, including register name and registration number, or state that the review was not registered.	5
	24b	Indicate where the review protocol can be accessed, or state that a protocol was not prepared.	5
	24c	Describe and explain any amendments to information provided at registration	N/A

Section and Topic	Item #	Checklist item	Location where item is reported
		or in the protocol.	
Support	25	Describe sources of financial or non-financial support for the review, and the role of the funders or sponsors in the review.	15
Competing interests	26	Declare any competing interests of review authors.	15
Availability of data, code and other materials	27	Report which of the following are publicly available and where they can be found: template data collection forms; data extracted from included studies; data used for all analyses; analytic code; any other materials used in the review.	15

Supplementary S3: Risk of bias summary for the included RCTs according to Risk of Bias 2 tool (RoB 2) [2]

Study	Bias from randomization process	Bias due to deviations from intended interventions	Bias due to missing outcome data	Bias in measurement of the outcomes	Bias in selection of the reported result	Overall risk of bias
Hart 2018	Low	Low	Low	Low	Low	Low
Diener 2019	Low	Low	Low	Low	Low	Low
Geisler 2024	Low	Low	Low	Low	Low	Low
Kamel 2024	Low	Low	Low	Low	Low	Low

Supplementary S4: Results of leave-one-out analysis for the primary efficacy outcomes

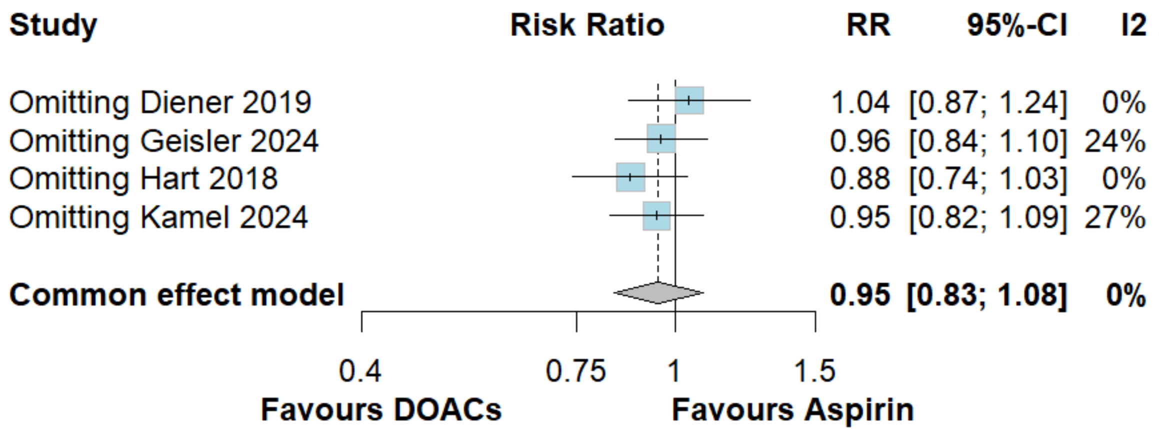

Supplementary S5A: Results of meta-analysis for ischemic stroke

	DOAC		Aspirin			Risk ratio
Study or Subgroup	Events	Total	Events	Total	Weight	M-H, Fixed, 95% CI
Diener 2019	172	2695	203	2695	49.4%	0.85 [0.70 , 1.03]
Geisler 2024	11	178	12	174	3.0%	0.90 [0.41 , 1.98]
Hart 2018	158	3609	156	3604	38.0%	1.01 [0.81 , 1.26]
Kamel 2024[a]	37	507	40	508	9.7%	0.93 [0.60 , 1.42]
**Total (95% CI)**		**6989**		**6981**	**100.0%**	**0.92 [0.80 , 1.05]**
Total events:	378		411			

Heterogeneity: Chi² = 1.42, df = 3 (P = 0.70); I² = 0%
Test for overall effect: Z = 1.23 (P = 0.22)
Test for subgroup differences: Not applicable

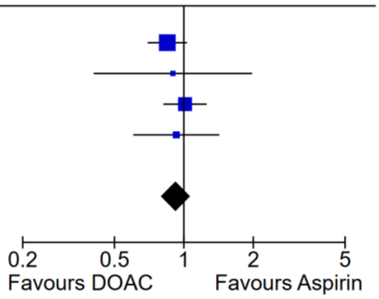

Footnotes
[a]Including systemic embolism

Supplementary S5B: Results of meta-analysis for hemorrhagic stroke

	DOAC		Aspirin			Risk ratio
Study or Subgroup	Events	Total	Events	Total	Weight	M-H, Random, 95% CI
Diener 2019	6	2695	7	2695	53.1%	0.86 [0.29 , 2.55]
Hart 2018	13	3609	2	3604	46.9%	6.49 [1.47 , 28.74]
**Total (95% CI)**		**6304**		**6299**	**100.0%**	**2.21 [0.29 , 16.69]**
Total events:	19		9			

Heterogeneity: Tau² = 1.69; Chi² = 4.82, df = 1 (P = 0.03); I² = 79%
Test for overall effect: Z = 0.77 (P = 0.44)
Test for subgroup differences: Not applicable

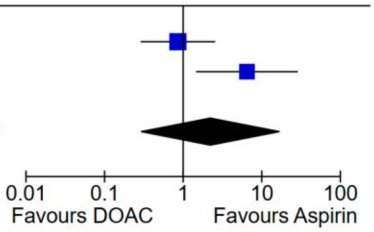

Supplementary S5C: Results of meta-analysis for systemic embolism

	DOAC		Aspirin			Risk ratio
Study or Subgroup	Events	Total	Events	Total	Weight	M-H, Fixed, 95% CI
Diener 2019	6	2695	11	2695	75.8%	0.55 [0.20 , 1.47]
Geisler 2024	0	178	1	174	10.4%	0.33 [0.01 , 7.95]
Hart 2018	1	3609	2	3604	13.8%	0.50 [0.05 , 5.50]
**Total (95% CI)**		**6482**		**6473**	**100.0%**	**0.52 [0.21 , 1.24]**
Total events:	7		14			

Heterogeneity: Chi² = 0.09, df = 2 (P = 0.95); I² = 0%
Test for overall effect: Z = 1.47 (P = 0.14)
Test for subgroup differences: Not applicable

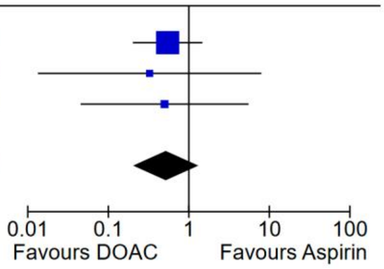

Supplementary S5D: Results of meta-analysis for myocardial infarction

	DOAC		Aspirin			Risk ratio
Study or Subgroup	Events	Total	Events	Total	Weight	M-H, Fixed, 95% CI
Diener 2019	23	2695	18	2695	40.9%	1.28 [0.69 , 2.36]
Geisler 2024	1	178	3	174	6.9%	0.33 [0.03 , 3.10]
Hart 2018	17	3609	23	3604	52.2%	0.74 [0.40 , 1.38]
**Total (95% CI)**		**6482**		**6473**	**100.0%**	**0.93 [0.61 , 1.42]**
Total events:	41		44			

Heterogeneity: Chi² = 2.38, df = 2 (P = 0.30); I² = 16%
Test for overall effect: Z = 0.33 (P = 0.74)
Test for subgroup differences: Not applicable

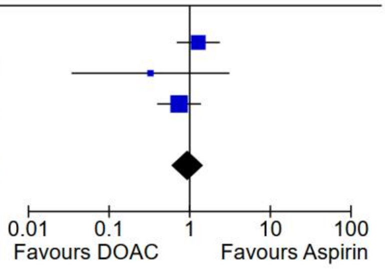

Supplementary S5E: Results of meta-analysis for all-cause mortality

Study or Subgroup	DOAC Events	DOAC Total	Aspirin Events	Aspirin Total	Weight	Risk ratio M-H, Fixed, 95% CI
Diener 2019	56	2695	58	2695	47.5%	0.97 [0.67 , 1.39]
Geisler 2024	3	178	4	174	3.3%	0.73 [0.17 , 3.23]
Hart 2018	65	3609	52	3604	42.6%	1.25 [0.87 , 1.79]
Kamel 2024	12	492	8	494	6.5%	1.51 [0.62 , 3.65]
**Total (95% CI)**		**6974**		**6967**	**100.0%**	**1.11 [0.87 , 1.42]**
Total events:	136		122			

Heterogeneity: Chi² = 1.73, df = 3 (P = 0.63); I² = 0%
Test for overall effect: Z = 0.87 (P = 0.38)
Test for subgroup differences: Not applicable

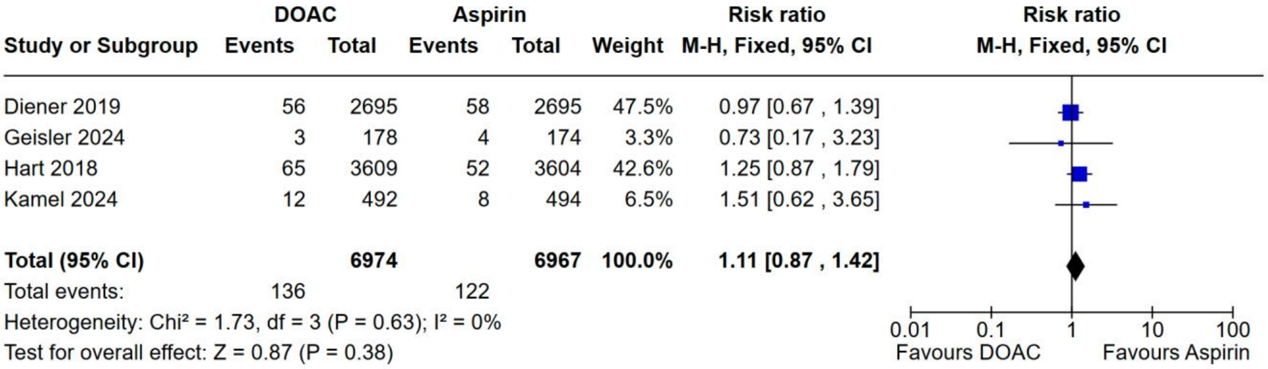

Supplementary S5F: Results of meta-analysis for death from cardiovascular events

Study or Subgroup	DOAC Events	DOAC Total	Aspirin Events	Aspirin Total	Weight	Risk ratio M-H, Fixed, 95% CI
Diener 2019	19	2695	24	2695	50.0%	0.79 [0.43 , 1.44]
Geisler 2024	1	178	1	174	2.1%	0.98 [0.06 , 15.51]
Hart 2018	34	3609	23	3604	47.9%	1.48 [0.87 , 2.50]
**Total (95% CI)**		**6482**		**6473**	**100.0%**	**1.12 [0.76 , 1.66]**
Total events:	54		48			

Heterogeneity: Chi² = 2.35, df = 2 (P = 0.31); I² = 15%
Test for overall effect: Z = 0.59 (P = 0.56)
Test for subgroup differences: Not applicable

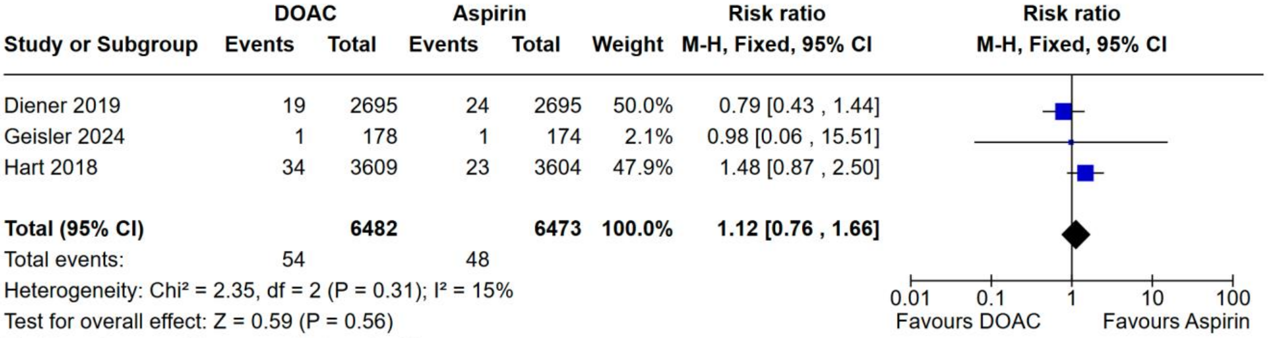

Supplementary S5G: Results of meta-analysis for the composite endpoint of non-fatal stroke, non-fatal myocardial infarction and cardiovascular death

Study or Subgroup	DOAC Events	DOAC Total	Aspirin Events	Aspirin Total	Weight	Risk ratio M-H, Fixed, 95% CI
Diener 2019[a]	207	2695	232	2695	52.5%	0.89 [0.75 , 1.07]
Geisler 2024[b]	13	178	15	174	3.4%	0.85 [0.42 , 1.73]
Hart 2018[c]	207	3609	195	3604	44.1%	1.06 [0.88 , 1.28]
**Total (95% CI)**		**6482**		**6473**	**100.0%**	**0.96 [0.85 , 1.10]**
Total events:	427		442			

Heterogeneity: Chi² = 1.80, df = 2 (P = 0.41); I² = 0%
Test for overall effect: Z = 0.55 (P = 0.58)
Test for subgroup differences: Not applicable

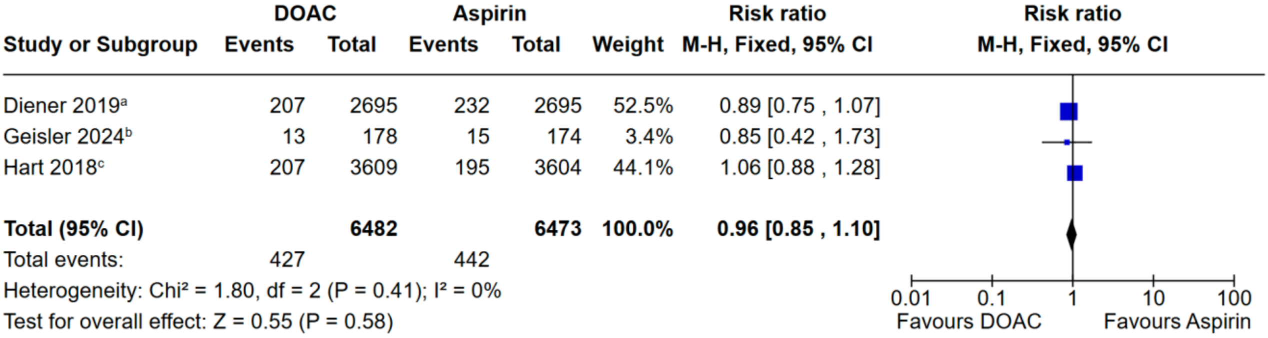

Footnotes

[a]Composite of non fatal stroke, non fatal myocardial infarction, or cardiovascular death
[b]Composite of any recurrent stroke, myocardial infarction or death from cardiovascular or unknown cause
[c]Composite of any recurrent stroke, myocardial infarction, death from cardiovascular causes or systemic embolism

Supplementary S6A: Results of leave-one-out analysis for meta-analysis of secondary efficacy outcomes “recurrent ischemic stroke”

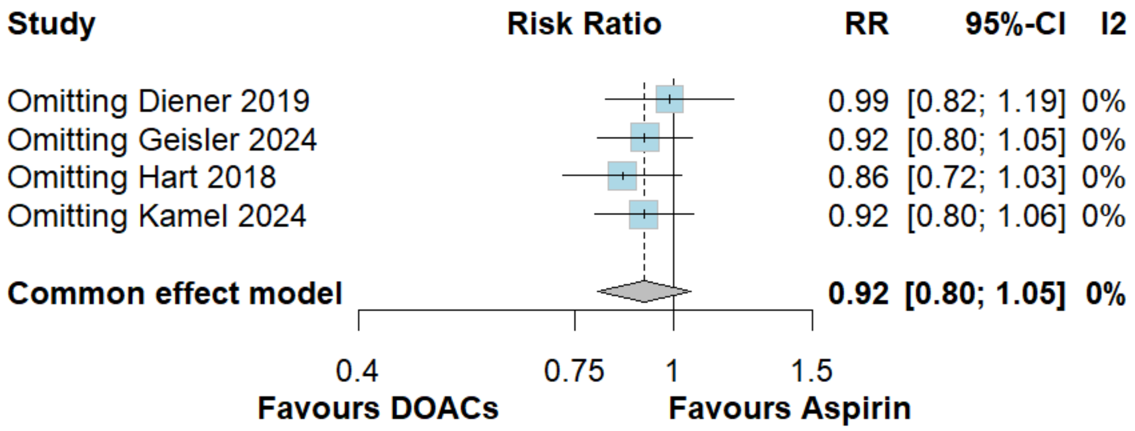

Supplementary S6B: Results of leave-one-out analysis for meta-analysis of secondary efficacy outcomes “systemic embolism”

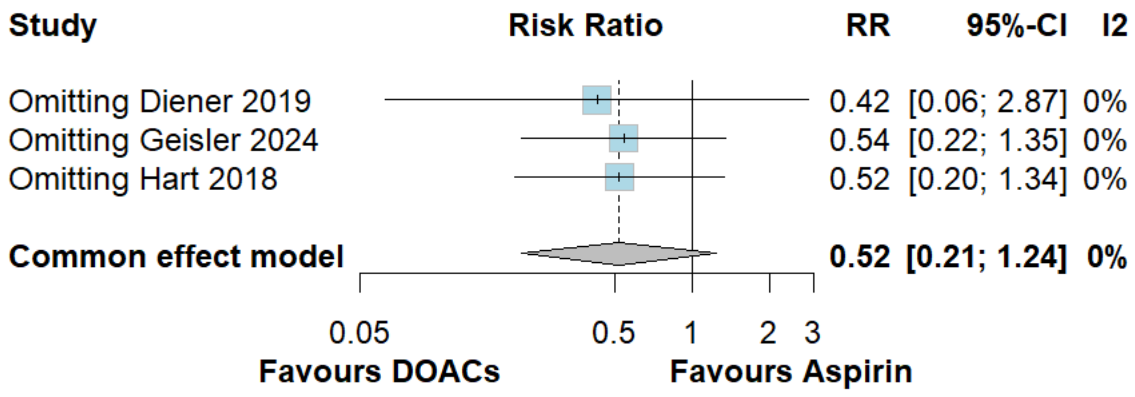

Supplementary S6C: Results of leave-one-out analysis for meta-analysis of secondary efficacy outcomes “myocardial infarction”

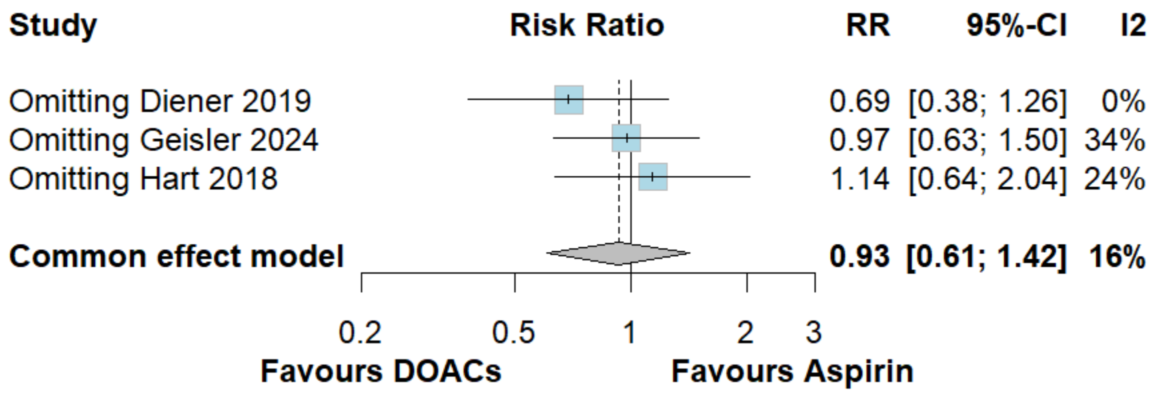

Supplementary S6D: Results of leave-one-out analysis for meta-analysis of secondary efficacy outcomes "all-cause mortality"

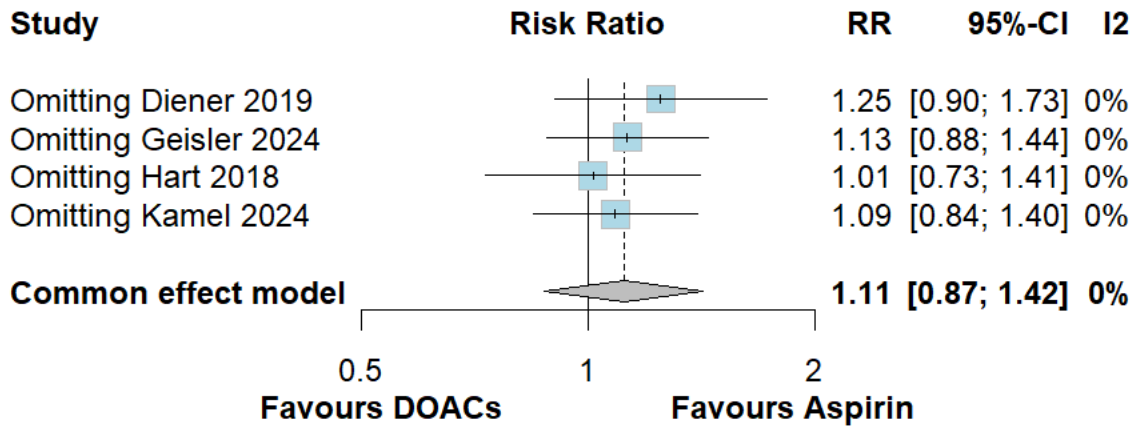

Supplementary S6E: Results of leave-one-out analysis for meta-analysis of secondary efficacy outcomes "death from cardiovascular events"

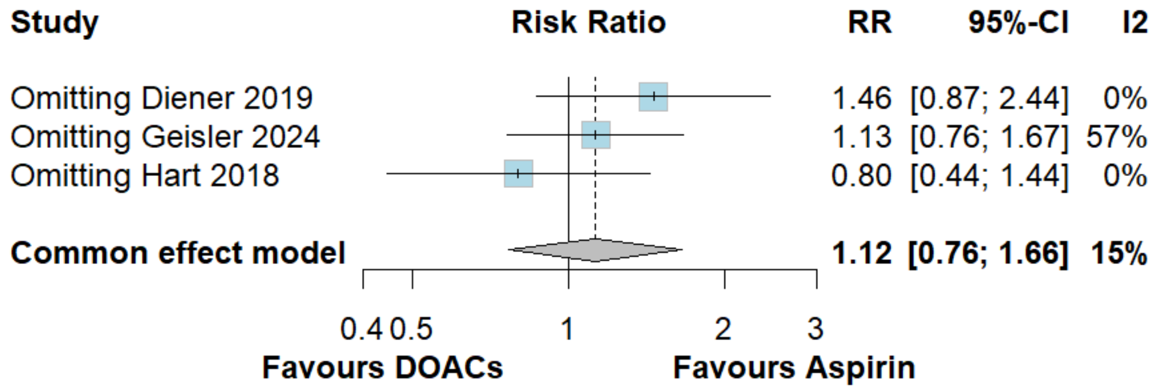

Supplementary S6F: Results of leave-one-out analysis for meta-analysis of secondary efficacy outcomes "non-fatal stroke, non-fatal myocardial infarction and cardiovascular death "

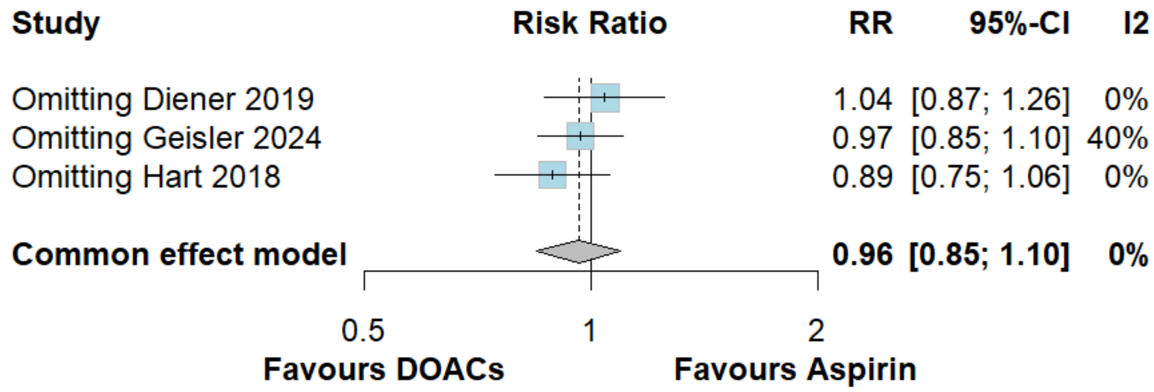

Supplementary S7A: Results of leave-one-out analysis for meta-analysis of primary safety outcomes "Major bleeding"

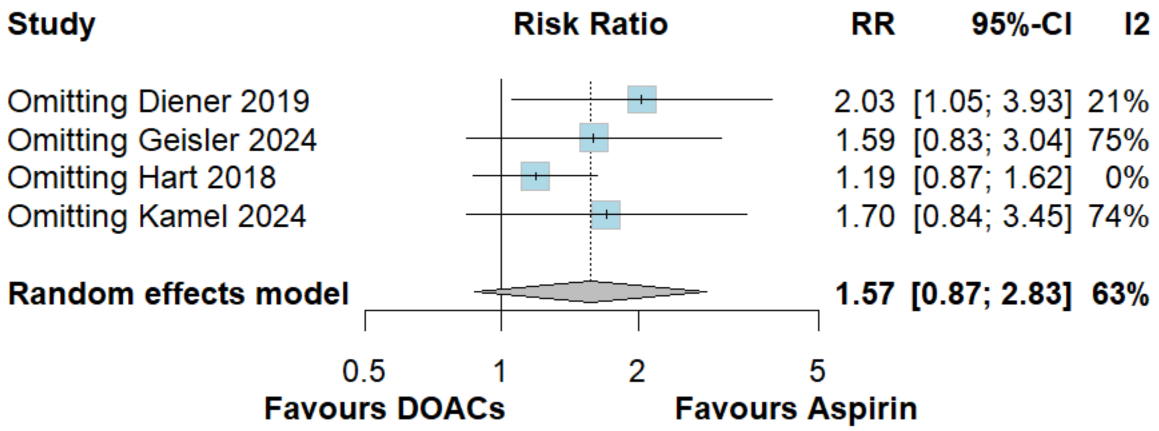

Supplementary S7B: Results of leave-one-out analysis for meta-analysis of primary safety outcomes "Clinically relevant non-major bleeding"

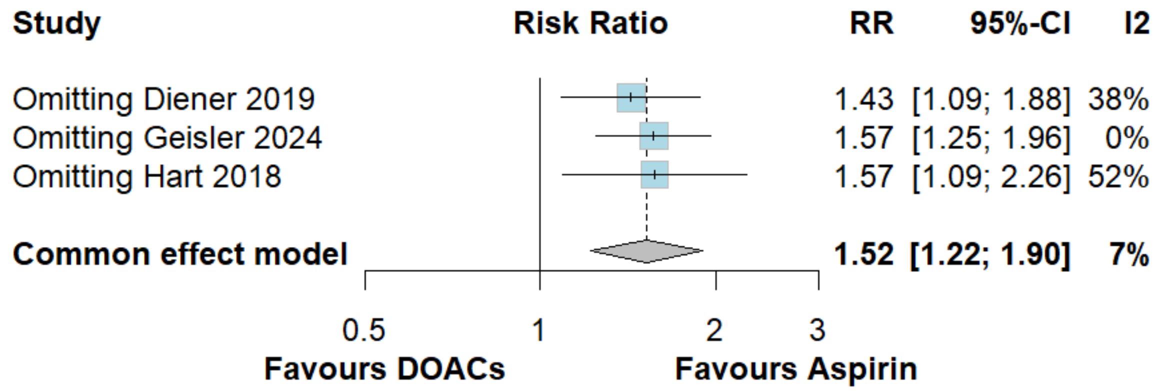

Supplementary S7C: Results of leave-one-out analysis for meta-analysis of primary safety outcomes "composite of major bleeding and clinically relevant non-major bleeding"

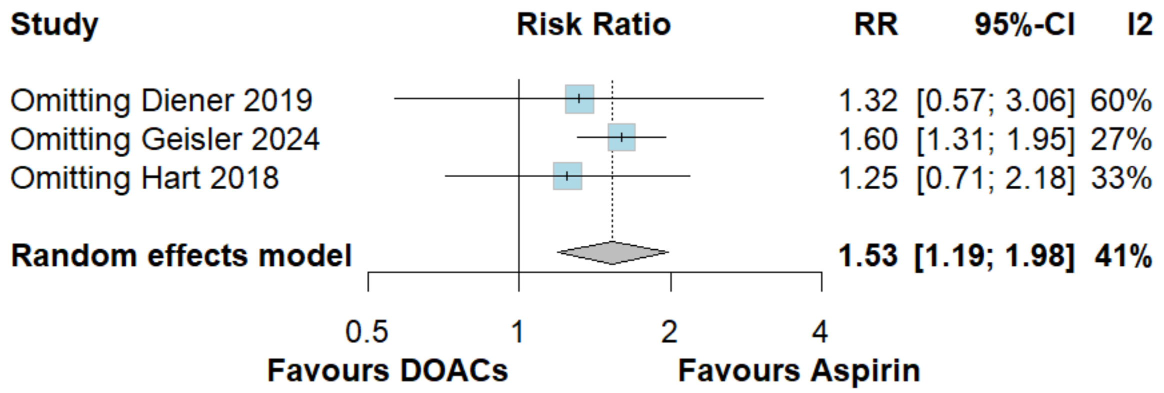

Supplementary S7D: Results of leave-one-out analysis for meta-analysis of primary safety outcomes "intracranial hemorrhage"

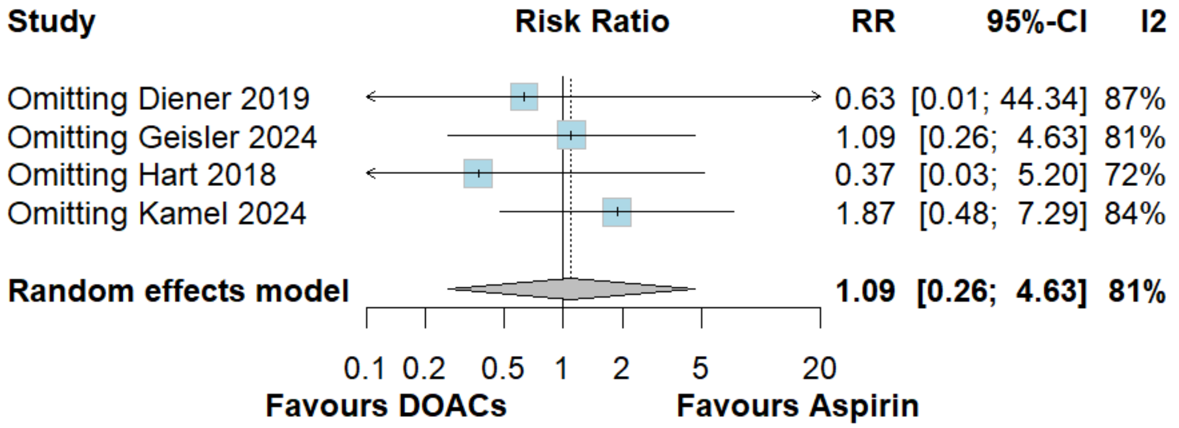

Supplementary S8A: Results of leave-one-out analysis for meta-analysis of subgroup analysis "age<65 years"

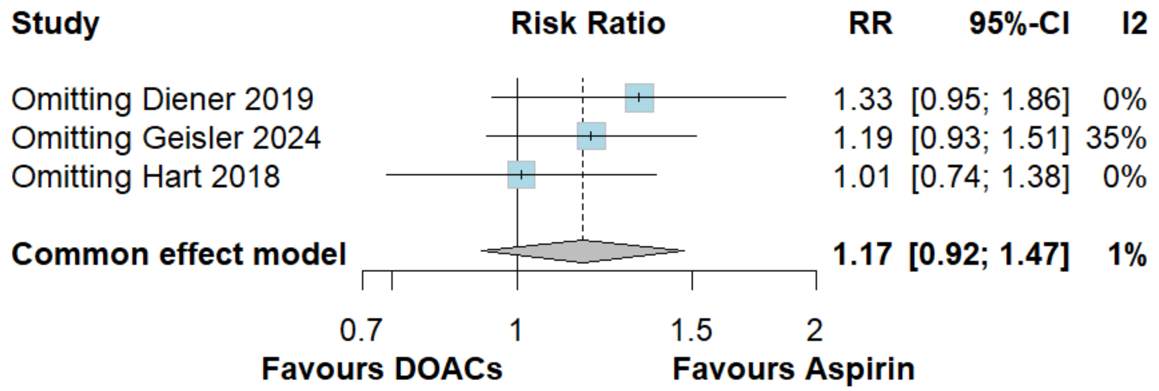

Supplementary S8B: Results of leave-one-out analysis for meta-analysis of subgroup analysis "age 65 to 74 years"

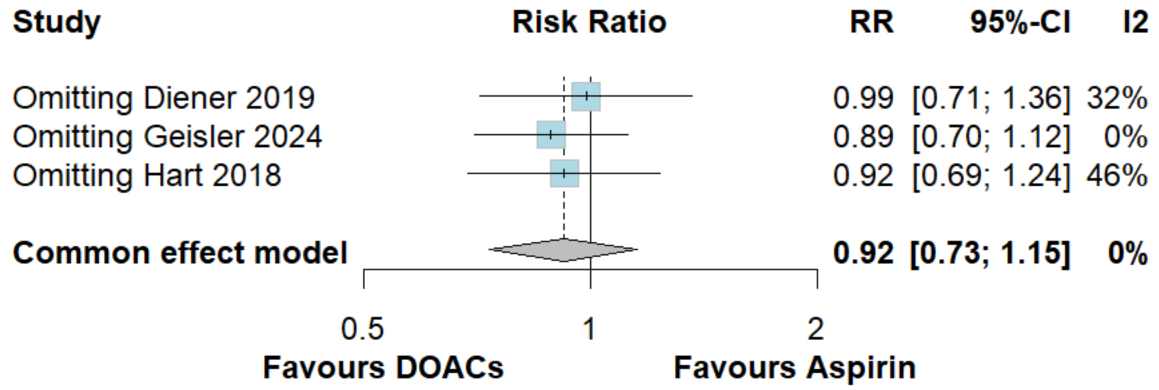

Supplementary S8C: Results of leave-one-out analysis for meta-analysis of subgroup analysis “age≥75 years”

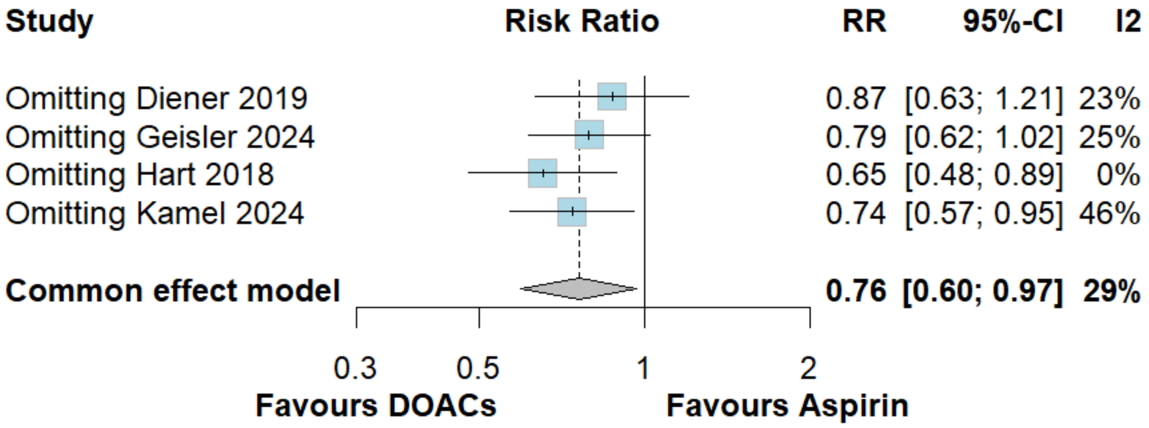

References

1. Page MJ, McKenzie JE, Bossuyt PM, et al (2021) The PRISMA 2020 statement: an updated guideline for reporting systematic reviews. BMJ 372:n71. https://doi.org/10.1136/bmj.n71
2. Sterne JAC, Savović J, Page MJ, et al (2019) RoB 2: a revised tool for assessing risk of bias in randomised trials. BMJ 366:l4898. https://doi.org/10.1136/bmj.l4898
